# Regulation and functional importance of human periodontal ligament mesenchymal stromal cells with various rates of CD146+ cells

**DOI:** 10.3389/fcell.2025.1532898

**Published:** 2025-03-07

**Authors:** Oliwia Miłek, Katharina Schwarz, Alma Miletić, Johanna Reisinger, Alexander Kovar, Christian Behm, Oleh Andrukhov

**Affiliations:** Competence Center for Periodontal Research, University Clinic of Dentistry, Medical University of Vienna, Vienna, Austria

**Keywords:** mesenchymal stem cells, stromal cells, periodontal ligament, periodontitis, CD146

## Abstract

**Introduction:**

Mesenchymal stromal cells (MSCs) with high expression of CD146 have superior properties for tissue regeneration. However, high variability in the rate of CD146+ cells among donors is observed. In this study, the possible reasons behind this variability in human periodontal ligament MSCs (hPDL-MSCs) were explored.

**Methods:**

hPDL-MSCs were isolated from 22 different donors, and rates of CD146+ cells were analyzed by flow cytometry. Furthermore, populations with various rates of CD146+ cells were isolated with magnetic separation. The dependency of cell proliferation, viability, cell cycle, and osteogenic differentiation on the rates of CD146+ cells was investigated. Besides, the effects of various factors, like cell density, confluence, and inflammatory environment on the CD146+ rate and expression were analyzed.

**Results:**

The rate of CD146+ cells exhibited high variability between donors, with the percentage of CD146+ cells ranging from 3% to 67%. Higher percentage of CD146+ cells was associated with higher proliferation, presumably due to the higher percentage of cells in the S-phase, and higher osteogenic differentiation potential. Prolonged cell confluence and higher cell seeding density led to the decline in the rate of CD146+ cells. The surface rate of CD146 in hPDL-MSCs was stimulated by the treatment with interleukin-1β and tumor necrosis factor-α, and inhibited by the treatment with interferon-γ.

**Conclusion:**

These results suggest that hPDL-MSCs with high rate of CD146+ cells are a promising subpopulation for enhancing the effectiveness of MSC-based regenerative therapies, however the rate of CD146 is affected by various factors, which must be considered for cell propagation and their potential application *in vivo*.

## 1 Introduction

For years, mesenchymal stromal cells (MSCs) have been researched as a promising remedy in tissue regeneration (Sagaradze et al., 2020), immunotherapy (Almeida-Porada et al., 2020) and chronic diseases (Huang et al., 2022). Those multipotent cells are defined by minimal criteria of plastic adherence, expression of specific surface markers (CD105, CD73, and CD90), lack of expression of hematopoietic and endothelial markers (CD11b, CD14, CD19, CD34, CD45, CD79a, and HLA-DR), and *in vitro* trilineage differentiation to osteoblasts, adipocytes, and chondrocytes (Dominici et al., 2006; Viswanathan et al., 2019; Renesme et al., 2025). Although MSCs are the recurrent cell type in cell-based therapies, the promising results of basic research and early-phase clinical trials do not translate to clinical applications (García-Bernal et al., 2021). The heterogeneity of MSCs, present at donor, tissue, and cellular levels, largely contributes to the inconsistencies in the outcomes of clinical trials. Moreover, the differences in cell culture and handling, such as expansion methods, can alter MSCs’ functionality (Galipeau and Sensébé, 2018).

Dental MSCs come from multiple sources within the oral cavity, such as dental pulp (Gronthos et al., 2000), periodontal ligament (Seo et al., 2004), gingiva (Zhang et al., 2009), apical papilla (Jo et al., 2007), exfoliated deciduous teeth (Miura et al., 2003), dental follicle (Morsczeck et al., 2005), and periapical cyst (Marrelli et al., 2013). The dental MSCs most commonly considered for clinical applications originate from dental pulp and periodontal ligament (Li et al., 2021; Song et al., 2023). Human periodontal ligament mesenchymal stromal cells (hPDL-MSCs) are particularly notable for their role in maintaining periodontal tissue homeostasis and regeneration. The therapeutic potential of hPDL-MSCs has favorable prospects, and the number of clinical trials using these cells in regenerative periodontology is increasing (Queiroz et al., 2021).

CD146 (also MCAM, MUC18, P1H12) is a cell surface glycoprotein, functioning as an adhesion molecule in cell-cell and cell-matrix interactions (Wang et al., 2020). CD146 is also a surface marker for MSCs isolated from multiple tissues, but unlike other markers such as CD73, CD90, or CD105, the expression of CD146 on MSCs is not uniform (Sorrentino et al., 2008; Wang and Yan, 2013). Within heterogeneous MSCs, the CD146+ cell subpopulation has been reported to have superior qualities in cells derived from bone marrow (Herrmann et al., 2019; Bowles et al., 2020), dental pulp (Matsui et al., 2018; Ma et al., 2021), periodontal ligament (Saito et al., 2013; Zhu et al., 2013; Cho et al., 2016) umbilical cord (Zhang et al., 2022; 2023), placenta (Ulrich et al., 2015), and other sources. CD146 is also attributed to clinical grade qualities of MSCs and high therapeutic potency (Bowles et al., 2020; Ma et al., 2021). In periodontal ligament, CD146+ cells have shown higher colony-forming efficiency, proliferative potential, and differentiation potential to osteoblasts and adipocytes (Saito et al., 2013; Zhu et al., 2013; Cho et al., 2016). In order to benefit from CD146+ cell features in the clinic, CD146+ hPDL-MSCs ought to be comprehensively characterized. The reasons behind CD146 heterogeneity need to be explained, and research should be carried out on CD146 stability during culturing and inflammatory response.

The aim of the present study was to explore the heterogeneity of CD146+ cell rate in hPDL-MSCs. In particular, we investigated how the rate of CD146+ hPDL-MSCs is changed during prolonged culture and upon stimulation with various inflammatory stimuli. When using original cell population and evaluating the rate of CD146 based on anti-CD146 antibody staining, we use term “CD146+” for positively-stained cells, and term “CD146−” for cells lacking surface CD146 staining.

**FIGURE 1:**
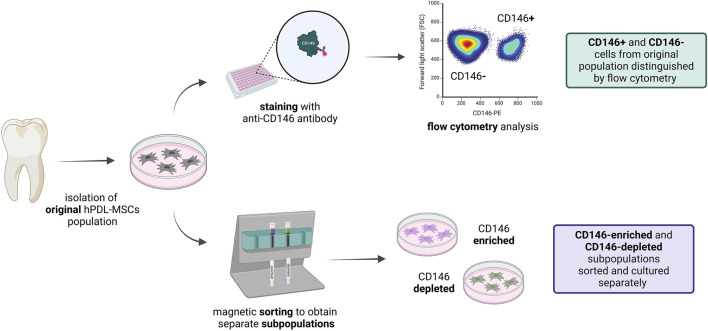
Graphical visualization of the model. hPDL-MSCs were isolated from molars and subcultured as explants. After cell outgrowth, hPDL-MSCs were used for experiments. Not sorted cells were stained with anti-CD146 antibody, and CD146+ and CD146− were evaluated with flow cytometry. hPDL-MSCs were also sorted using magnetic separation, and obtained CD146-enriched and CD146-depleted subpopulations were further cultured separately, for use in subsequent experiments. This figure was created with BioRender.com.

Furthermore, we isolated subpopulations of hPDL-MSCs with different rates of CD146+ cells using magnetic beads, and investigated their basic properties, including proliferation, osteogenic differentiation, and the stability of CD146 rate during passaging. We use term “CD146-enriched” for positively-selected CD146+ cells, and term “CD146-depleted” for cells after negative selection. Graphical explanation of the model is presented in Figure 1.

## 2 Materials and methods

### 2.1 Isolation of hPDL-MSCs

Human periodontal ligament mesenchymal stromal cells (hPDL-MSCs) were isolated from the periodontal ligaments of healthy molars removed due to orthodontic procedures. In total, the 22 donors were of both sexes (13 females and 9 males) and aged between 18 and 36. Cell isolation protocol was approved by the Ethics Committee affiliated with the Medical University of Vienna (No. 1079/2019). All the procedures followed the “Good Scientific Practice” regulations of the Medical University of Vienna and the ethical principles outlined in the Declaration of Helsinki, and the participants gave their written consent. The isolation procedure has been previously described (Miłek et al., 2023), and stem cell features were validated for cells isolated with this method (Behm et al., 2024b). Briefly, the periodontal ligament was carefully scraped from the tooth and plated on a tissue culture plate (TPP, Trasadingen, Switzerland), then supplemented with culture medium (Dulbecco’s Modified Eagle Medium High Glucose (DMEM HG; supplemented with 4.5 g/mL glucose, L-glutamine), 10% of fetal bovine serum (FBS), 100 I.U./mL penicillin and 100 μg/mL streptomycin (all from Capricorn Scientific, Ebsdorfergrund, Germany). Isolated cells were cultured at 37°C with 5% CO_2_ under humidified conditions in culture medium. Cells were detached with accutase (Capricorn Scientific, Ebsdorfergrund, Germany), passaged once they reached 80% confluence, and used until passage 7. The list of donors used for certain experiments can be found in Supplementary Table 1.

### 2.2 Surface CD146 staining and flow cytometry

The cells were incubated with PE-conjugated anti-CD146 monoclonal antibody (clone P1H12; Thermo Fisher Scientific, Waltham, MA, United States) at a dilution of 1:100 in staining buffer (1× PBS (Gibco, Waltham, MA, United States), 30% w/v bovine serum albumin (BSA; Capricorn Scientific, Ebsdorfergrund, Germany), and 0.09% w/v sodium azide (Sigma-Aldrich, St. Louis, MO, United States) for 30 min at room temperature. After washing twice with a staining buffer without antibodies, cells were analyzed with Attune NxT Flow Cytometer (Invitrogen, Waltham, MA, United States) using Attune NxT Flow Cytometer Software v3.1.2. Doublets were excluded, and the percentage of cells with positive (CD146+) and negative (CD146−) surface rate of CD146 was assessed, taking unstained cells as a reference. Mean fluorescent intensity (MFI) measurement was used to quantify surface CD146 expression. MFI was calculated from CD146+ cells, through an average fluorescence intensity of the cell population, and normalized to control cells. All measurements were performed using Attune NxT Flow Cytometer Software v3.1.2.

### 2.3 Rate of CD146+ cells during prolonged culture depending on the initial seeding density

hPDL-MSCs were seeded in 6-well cell culture plates (TPP, Trasadingen, Switzerland) at densities 5 × 10^3^, 1 × 10^4^, and 2.5 × 10^4^ cells per cm^2^ in 500 µL of culture medium (DMEM HG + 10% FBS + 100 I.U./mL penicillin and 100 μg/mL streptomycin). Donors with intermediate (around 50%) CD146+ rate were used for those experiments, to allow for either an increase or decrease in CD146 percentage. Separate plates were prepared for each day of the experiment, and the medium was changed on day 3. On days 1–7, cells were washed with 1× PBS and detached into single-cell suspension with accutase. 1 × 10^5^ cells were evaluated for CD146+ rate with flow cytometry, as described above.

### 2.4 Stimulation of hPDL-MSCs with inflammatory cytokines

hPDL-MSCs were seeded in 24-well cell culture plates (TPP, Trasadingen, Switzerland) at a density of 4 × 10^4^ cells per cm^2^ in 500 µL of culture medium (DMEM HG + 10% FBS + 100 I.U./mL penicillin and 100 μg/mL streptomycin). After 24 h, cells were washed with 1× PBS and treated with inflammatory cytokines in a medium without FBS (DMEM HG + 100 I.U./mL penicillin and 100 μg/mL streptomycin). The recombinant cytokines (all from PeproTech, London, United Kingdom) were used at the following concentrations: 0.5 and 5 ng/mL interleukin (IL)-1β, 10 and 100 ng/mL interferon (IFN)-γ, and 1 and 10 ng/mL tumor necrosis factor (TNF)-α. The cytokines and their concentrations were selected based on our previous studies (Behm et al., 2020b). The cells were treated for 24 h (for ELISA) and 48 h (for ELISA and flow cytometry). After the treatment, cell culture supernatants were collected and frozen at −80°C, and cells were rinsed with PBS. For each group, the cells from two wells per donor were combined for staining. Then, the cells were fixed using FIX & PERM Cell Permeabilization Kit (Invitrogen, Waltham, MA, United States) following the manufacturer’s protocol and the percentage of CD146+ cells was analyzed with flow cytometry, as described above.

Concentrations of soluble CD146 in cell culture supernatants were detected using a Human MCAM/CD146 ELISA Kit (Invitrogen, Waltham, MA, United States). Briefly, 100 µL of undiluted samples were added to the wells and incubated overnight at 4°C with gentle shaking. Then, the manufacturer’s protocol was followed. The absorbance of the final solution was measured at a wavelength of 450 nm using Synergy HTX Multimode Reader (BioTek, Winooski, VT, United States). Sample concentrations were calculated based on an extrapolated standard curve using Arigo’s ELISA Calculator (arigo Biolaboratories, Hsinchu, Taiwan).

### 2.5 Isolation of hPDL-MSCs subpopulations with different levels of CD146+ rates using magnetic beads

The isolation of hPDL-MSCs with different levels of CD146+ rate was accomplished using Dynabeads FlowComp Flexi Kit (Invitrogen, Waltham, MA, United States) following the manufacturer’s protocol. Donors with intermediate (around 50%) CD146+ rate were used for those experiments, to allow for either an increase or decrease in CD146+ rate. Briefly, cells were washed with 1× PBS and detached into single-cell suspension with accutase. 1 × 10^6^ cells were incubated with previously biotinylated CD146 monoclonal antibody (clone P1H12; eBioscience, Frankfurt am Main, Germany) for 30 min at 4°C. Afterward, cells were washed with isolation buffer [1× Ca^2+^ and Mg^2+^ free PBS (Gibco, Waltham, MA, United States), 0.1% w/v BSA and 2 mM EDTA (Sigma-Aldrich, St. Louis, MO, United States)] and incubated with streptavidin-bound magnetic beads for 30 min at 4°C with gentle shaking. Then, the antibody-coupled cells were captured by magnetic beads, based on biotin-streptavidin binding, and the complexes were separated with the use of DynaMag-5 Magnet (Invitrogen, Waltham, MA, United States). The negative fraction was collected and designated as cells with lower CD146+ rate (CD146-depleted hPDL-MSCs). The leftover magnet-bound cells were incubated with a release buffer for 10 min at 4°C with gentle shaking, allowing release of the beads from the cells. Subsequently, those cells were once again separated with the magnet, and the fraction of bead-free cells was collected. These cells were designated as those with higher rate of CD146+ (CD146-enriched hPDL-MSCs). To evaluate isolation purity, 5 × 10^4^ cells from both CD146-enriched and CD146-depleted subpopulations were stained with a secondary antibody (Goat anti-Mouse IgG1 Cross-Adsorbed Secondary Antibody; Invitrogen, Waltham, MA, United States), which targets mouse IgG1 of previously used primary antibody, independently of performed biotinylation. The secondary antibody was used at a concentration of 1:800 in a staining buffer (1× PBS, 30% w/v BSA, 0.09% w/v sodium azide) for 30 min at room temperature. The samples were analyzed with flow cytometry, as described above. Only donors for whom the difference in percentage of CD146+ cells was higher than 30% between CD146-enriched and CD146-depleted subpopulations were used in further experiments. CD146-enriched and CD146-depleted subpopulations were cultured similarly to the protocols described above for the heterogeneous hPDL-MSCs. Before every experiment, CD146-enriched and CD146-depleted subpopulations were analyzed via flow cytometry as described above to ensure a minimum of 30% difference in the rate of CD146+ cells.

### 2.6 Osteogenic differentiation and Alizarin Red staining

CD146-enriched and CD146-depleted hPDL-MSCs were seeded separately onto 24-well cell culture plates at a density of 2 × 10^4^ cells per cm^2^ in 500 µL of culture medium (DMEM HG + 10% FBS + 100 I.U./mL penicillin and 100 μg/mL streptomycin). The cells were cultured for 1 week, and on day 7, osteogenic differentiation was induced. The cells were washed with 1× PBS, and culture medium was replaced by osteogenic medium composed of αMEM (Capricorn Scientific, Ebsdorfergrund, Germany), 10% FBS, 100 I.U./mL penicillin, 100 μg/mL streptomycin, 0.1 μM dexamethasone, 50 μM ascorbic acid 2-phosphate, and 10 mM β-glycerophosphate (all from Sigma-Aldrich, St. Louis, MO, United States). Cells in some wells were further cultured in the culture medium and served as a negative control. All cells were cultured for 21 days in the corresponding media, with half of the media volume exchanged every 3–4 days.

On day 21 after the start of osteogenic differentiation, the cells were stained with Alizarin Red to visualize calcium deposits. The cells were washed with 1× PBS and fixed with ice-cold ethanol for 45 min at room temperature, followed by washing with distilled H_2_O (dH_2_O). Each well was submerged in 500 µL of 1% w/v Alizarin Red solution (ARS; Sigma-Aldrich, St. Louis, MO, United States), prepared in dH_2_O and filtered through a 0.45 μm filter. After 45 min of incubation at room temperature, ARS was removed, and wells were thoroughly washed with dH_2_O. For quantification, ARS was extracted with 500 µL per well of 10% w/v cetylpyridinium chloride solution (CPC; Sigma-Aldrich, St. Louis, MO, United States) in dH_2_O. After 45-min incubation at room temperature with gentle shaking, the solution was transferred to a 96-well plate, and absorbance was measured at a wavelength of 405 nm using the Synergy HTX Multimode Reader (BioTek, Winooski, VT, United States). CD146-enriched and CD146-depleted hPDL-MSCs from each donor were cultured in duplicates, and absorbance was measured in technical triplicates.

### 2.7 Cell cycle analysis

The cell cycle analysis was carried out using DAPI stain (Sigma-Aldrich, St. Louis, MO, United States), which intercalates between double-stranded DNA (dsDNA) and allows the distinction of different cell cycle phases based on fluorescence intensity of dsDNA content (Darzynkiewicz et al., 2017). The non-sorted hPDL-MSCs, as well as sorted CD146-enriched and CD146-depleted subpopulations, were used in the experiments. Non-confluent hPDL-MSCs in the growth stage were washed with 1× PBS and detached into single-cell suspension with accutase. Not sorted cells were stained with PE-CD146 monoclonal antibody (clone P1H12; Thermo Fisher Scientific, Waltham, MA, United States) at a dilution of 1:100 in a staining buffer (1× PBS, 30% w/v BSA, 0.09% w/v sodium azide) for 30 min at room temperature. Then, both not sorted cells and CD146-enriched and depleted hPDL-MSCs were fixed and permeabilized using FIX & PERM Cell Permeabilization Kit (Invitrogen, Waltham, MA, United States), following the manufacturer’s protocol. The cells were stained with DAPI diluted to 1:100 in Permeabilization Medium from the above-mentioned kit for 20 min at room temperature. Cell cycle analysis was performed using the Attune NxT Flow Cytometer (Invitrogen, Waltham, MA, United States). After doublet exclusion, not sorted cells were gated into CD146+ and CD146− groups based on CD146-positivity in comparison to unstained cells. Analysis of DNA content was performed in FCS Express Flow Cytometry Analysis Software (*De Novo* Software, Pasadena, CA, United States) using the “Multicycle DNA” feature, comparing cell cycle phases (Gap 1 - G1, synthesis - S, Gap 2 - G2, and mitosis - M) between CD146+ and CD146−, as well as CD146-enriched and CD146-depleted hPDL-MSCs.

### 2.8 Viability of CD146-enriched and CD146-depleted subpopulations

CD146-enriched and CD146-depleted subpopulations of hPDL-MSCs were isolated and outgrown in cell culture conditions to obtain higher cell numbers. The cells were seeded in 24-well cell culture plates at densities: 5 × 10^3^, 2 × 10^4^, and 5 × 10^4^ cells per cm^2^ in 500 µL of culture medium (DMEM HG + 10% FBS + 100 I.U./mL penicillin and 100 μg/mL streptomycin). On days 1, 2, 4, and 7 post-seeding, the Cell Counting Kit-8 (CCK-8; Dojindo Laboratories, Japan) was used to assess cell viability. This assay measures the activity of all cell dehydrogenases and is not toxic to the cells (Chamchoy et al., 2019). Following the manufacturer’s protocol, 50 µL of CCK-8 solution was added to each well and incubated for 2 h at 37°C with 5% CO_2_ under humidified conditions in the dark. Afterward, aliquots of the developed solution were transferred to a 96-well plate, and absorbance was measured at a wavelength of 450 nm. The media were changed and the cell were cultured. The obtained optical density (OD) for days 2, 4, and 7 was divided by the OD measured on day 1, giving “ratio to day 1”.

### 2.9 Statistical analysis

Each experiment was carried out with hPDL-MSCs from at least 4 different donors, with either 2 or 3 technical replicates. The statistical analysis, as well as graph production, was performed with GraphPad Prism 8 (Dotmatics, Boston, MA, United States). A Shapiro–Wilk test was used to evaluate the normal distribution of the data. Comparison between CD146-enriched and CD146-depleted, and CD146+ and CD146− hPDL-MSCs was carried out using either a paired t-test for normally distributed data or a Wilcoxon signed-rank test in other cases. For comparison of multiple groups, one-way or repeated measures (RM) ANOVA with *post hoc* Tukey were used. Statistically significant data were considered to have *p* values ≤ 0.05, and are reported on the graphs.

## 3 Results

### 3.1 Rate of CD146+ cells varies between hPDL-MSCs donors, regardless of passage and sex

**FIGURE 2:**
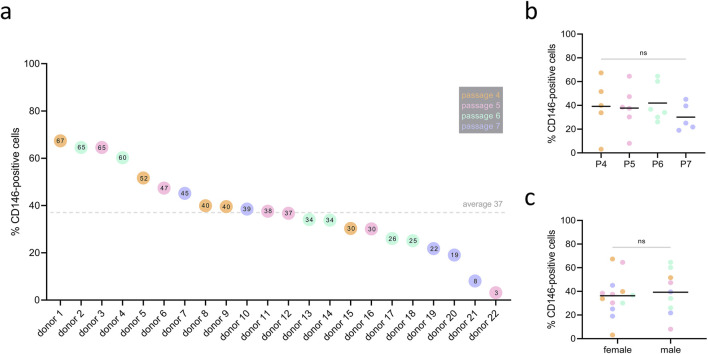
Rate of CD146+ cells among different hPDL-MSCs’ donors. hPDL-MSCs from 22 donors at passages 4–7 were stained with PE conjugated anti-CD146 antibody and the percentage of CD146+ cells was determined by flow cytometry. Data are presented as separate points for each donor, sorted by descending percentage of CD146+ cells **(A)**. The value inside of the points represents the percentage of CD146+ cells in an individual donor. The dotted line represents the mean value of 37% surface rate of CD146+ among donors. CD146+ rate was investigated in correlation to passage (P), but no statistically significant differences were observed **(B)** with one-way ANOVA. Similarly, CD146+ rate did not differ between female and male donors **(C)** using unpaired t-test. Color of the points represent passage number, with the legend listed in grey box (P4 – orange, P5 – pink, P6 – blue, P7 - purple).

The percentage of CD146+ cells among the different donors is shown in Figure 2A. Between 22 cell donors, large differences in the surface rate of CD146 were observed: the percentage of CD146+ cells ranged from 3% to 67%, with a mean of 37% ± 17% (SD), and a median of 38%. The passage of cells (4, 5, 6, 7) did not affect surface CD146+ rate among donors (Figure 2B). The surface CD146+ rate was independent of sex, with 36% ± 17% (SD) and a median of 37% rate in females, and 39% ± 19% (SD) and a median of 40% rate in males (Figure 2C).

### 3.2 Decreased rate of CD146+ cells during prolonged culture of hPDL-MSCs depends on the initial seeding density

**FIGURE 3:**
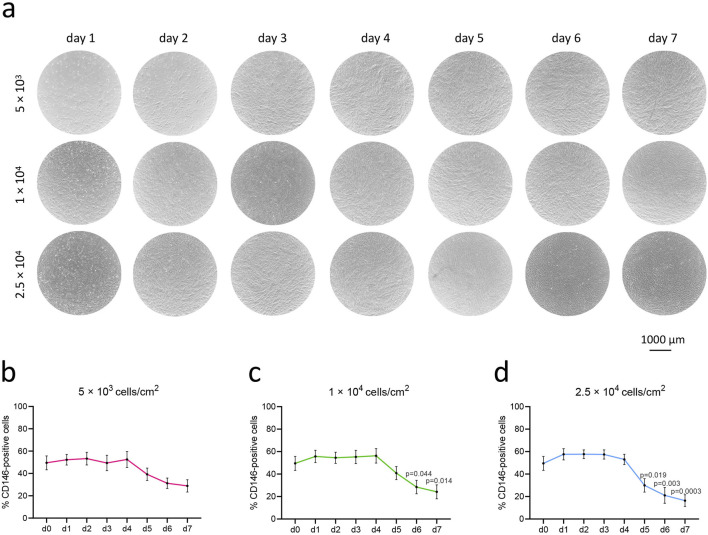
Rate of CD146+ cells in hPDL-MSCs upon over-time culture in various growing densities. hPDL-MSCs (n = 4) were seeded onto cell culture plates at different densities: 5 × 10^3^ cells/cm^2^, 1 × 10^4^ cells/cm^2^, and 2.5 × 10^4^ cells/cm^2^, and cultured for 7 days. Representative microscopic pictures from one donor were taken with the Echo Revolve microscope, with objective ×4 **(A)**. The graphs **(B–D)** represent the percentages of CD146+ cells during consecutive days, presented as mean ± SEM of 4 different donors. P values obtained with one-way ANOVA and *post hoc* Tukey tests are given for significantly different results in comparison to day 1 (d1).

Changes in the percentage of CD146+ hPDL-MSCs seeded at densities of 5 × 10^3^, 1 × 10^4^, and 2.5 × 10^4^ cells per cm^2^ and cultured for 7 days are shown in Figure 3. Each day, the confluence was visually monitored under the microscope, and the percentage of CD146+ cells was measured. By day 2, cells from the highest density group reached 100% confluence, followed by cells from the middle seeding density group reaching complete confluence on day 3, and cells from the lowest density group on day 4 (Figure 3A). Starting from day 5, CD146+ rate gradually declined in all groups, but the degree of this decline depended on the initial seeding density. The loss of surface CD146+ rate in cells seeded at the lowest density was not significant in comparison to day 1 (Figure 3B). Cells seeded at the middle density had a significant loss in CD146+ rate on days 6 and 7 in comparison to day 1 (Figure 3C); the percentage of CD146+ cells declined from 56% (day 1) to 24% (day 7). Cells seeded at the highest density had a significant loss in CD146+ rate on days 5, 6, and 7 compared to day 1 (Figure 3D); the percentage of CD146+ cells declined from 58% (day 1) to 16% (day 7).

### 3.3 Rate of CD146+ cells and surface CD146 expression in hPDL-MSCs changes upon treatment with inflammatory cytokines

**FIGURE 4:**
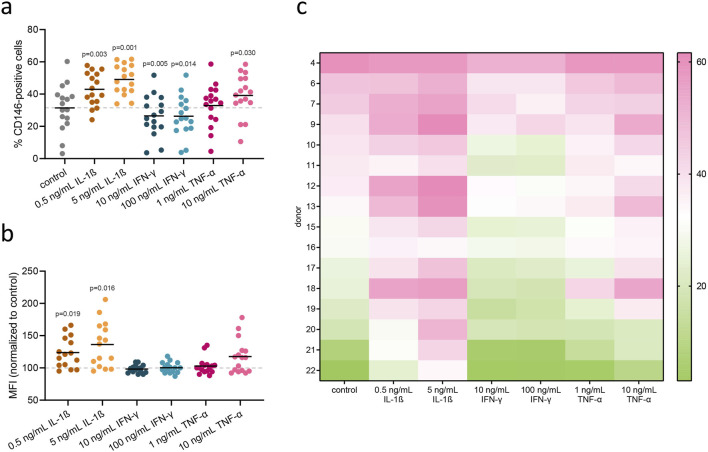
Flow cytometry analysis of surface CD146 in hPDL-MSCs upon treatment with inflammatory cytokines. hPDL-MSCs were stimulated for 48 h with inflammatory cytokines: IL-1β (0.5 and 5 ng/mL), IFN-γ (10 and 100 ng/mL), and TNF-α (1 and 10 ng/mL). Afterward, surface CD146 was stained with a monoclonal antibody and detected via flow cytometry. Each dot within a group presents a rate of CD146+ cells in different donor (n = 16), with mean values represented by the line **(A)**. MFI values, normalized to untreated control, are presented in the same manner **(B)**. P values obtained using RM one-way ANOVA with *post hoc* Tukey are given for statistically significant differences compared to unstimulated control cells. Rate of CD146+ cells for each of 16 donors are presented in the form of a heatmap **(C)**, measured across different treatment conditions (columns). Each cell’s color intensity represents the magnitude of response, with pink indicating higher values and green indicating lower values, as shown by the color bar.

hPDL-MSCs were stimulated for 48 h with inflammatory cytokines at different concentrations: IL-1β (0.5 and 5 ng/mL), IFN-γ (10 and 100 ng/mL), and TNF-α (10 and 100 ng/mL). Statistically significant changes in the percentage of CD146+ cells were observed in almost all treatments (Figures 4A, C), as compared to unstimulated cells. Stimulation with IL-1β at both concentrations, and with TNF-α at a concentration of 10 ng/mL, increased the percentage of CD146+ hPDL-MSCs compared to the unstimulated control. Conversely, stimulation with IFN-γ at both concentrations decreased the percentage of CD146+ cells compared to unstimulated control. Surface expression of CD146, based on MFI measurements, was significantly upregulated upon treatment with both concentrations of IL-1β, and an elevated tendency was observed at a higher concentration of TNF-α (Figure 4B).

CD146 concentrations in culture medium were evaluated after 24 and 48 h post-stimulation with IL-1β (5 ng/mL), IFN-γ (100 ng/mL), and TNF-α (100 ng/mL). After 24 h, conditioned media from cells treated with IFN-γ had significantly lower levels of soluble CD146 (sCD146) in comparison to unstimulated control, whereas sCD146 levels were significantly higher than the unstimulated group in media from cells treated with TNF-α (Supplementary Figure 2A). After 48 h, sCD146 levels in conditioned media from cells treated with IFN-γ were significantly lower in comparison to media from cells treated with IL-1β and TNF-α (Supplementary Figure 2B).

### 3.4 CD146-enriched hPDL-MSCs have higher osteogenic potential than CD146-depleted subpopulation, following magnetic separation

**FIGURE 5:**
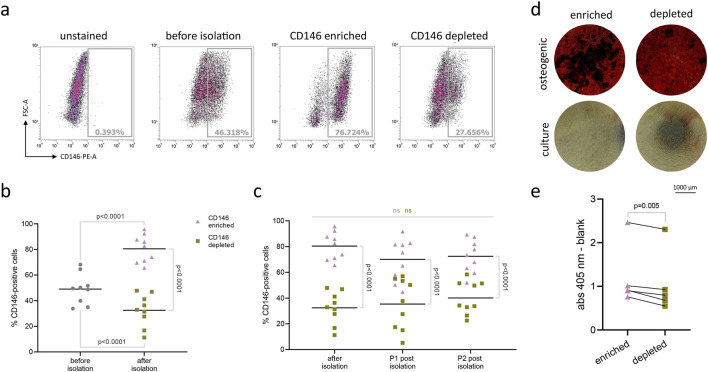
Isolation and osteogenic differentiation of CD146-enriched and CD146-depleted subpopulations of hPDL-MSCs. Original subpopulations of hPDL-MSCs were isolated using magnetic beads into distinct subpopulations expressing surface CD146 at high and low levels (n = 9). Flow cytometry results of one representative donor appear on graph **(A)**. Results are presented on graph **(B)**, with mean values represented by the line. Isolated subpopulations were significantly higher or lower in surface CD146+ rate than original populations (RM one-way ANOVA with *post hoc* Tukey). Isolated subpopulations were further cultured, and surface CD146+ rate was checked at each passage **(C)** to ensure significant differences between them (one-way ANOVA with *post hoc* Tukey). The mean values are represented by the line. Isolated subpopulations from 5 donors were grown on cell culture plates and maintained in either osteogenic or culture media as control. After 21 days, cells from both groups were stained with Alizarin Red dye to visualize calcium deposits. Results from one representative donor were pictured under the inverted Echo Revolve microscope, with objective ×4 **(D)**. Later, the dye was extracted using 10% w/v cetylpyridinium chloride solution, and the optical density was measured at 405 nm **(E)**. Paired t-test was used to compare the optical density values between different subpopulations.

After magnetic sorting, the rate of CD146+ cells was evaluated with flow cytometry as shown in Figure 5A. The collective data of CD146 rates in enriched and depleted subpopulations from 9 donors is shown in Figure 5B. The percentage of CD146+ cells was 49% ± 12% (SD) in the original cell population, 80% ± 11% in the CD146-enriched subpopulation, and 32% ± 13% in the CD146-depleted subpopulation (Figure 5B). During the subsequent 2 passages, the difference between enriched and depleted subpopulations was maintained without any statistically significant changes (Figure 5C). However, in subsequent passages, a small decrease in CD146+ rate was observed in CD146-enriched subpopulation, and a small increase in CD146+ rate was observed in CD146-depleted subpopulation.

The osteogenic differentiation of hPDL-MSCs subpopulations with different levels of surface CD146 is shown in Figure 5. Among 5 cell donors, the CD146-enriched subpopulation had significantly greater levels of osteogenic differentiation than the CD146-depleted subpopulation as suggested by an Alizarin Red staining (Figure 5D). Quantitative analysis revealed significantly higher optical density values after dye extraction in the enriched subpopulation, compared to depleted (Figure 5E).

### 3.5 When cultured in lower density, CD146-enriched hPDL-MSCs have a faster proliferation rate than CD146-depleted subpopulation, as assessed by their viability and cell cycle analysis

**FIGURE 6:**
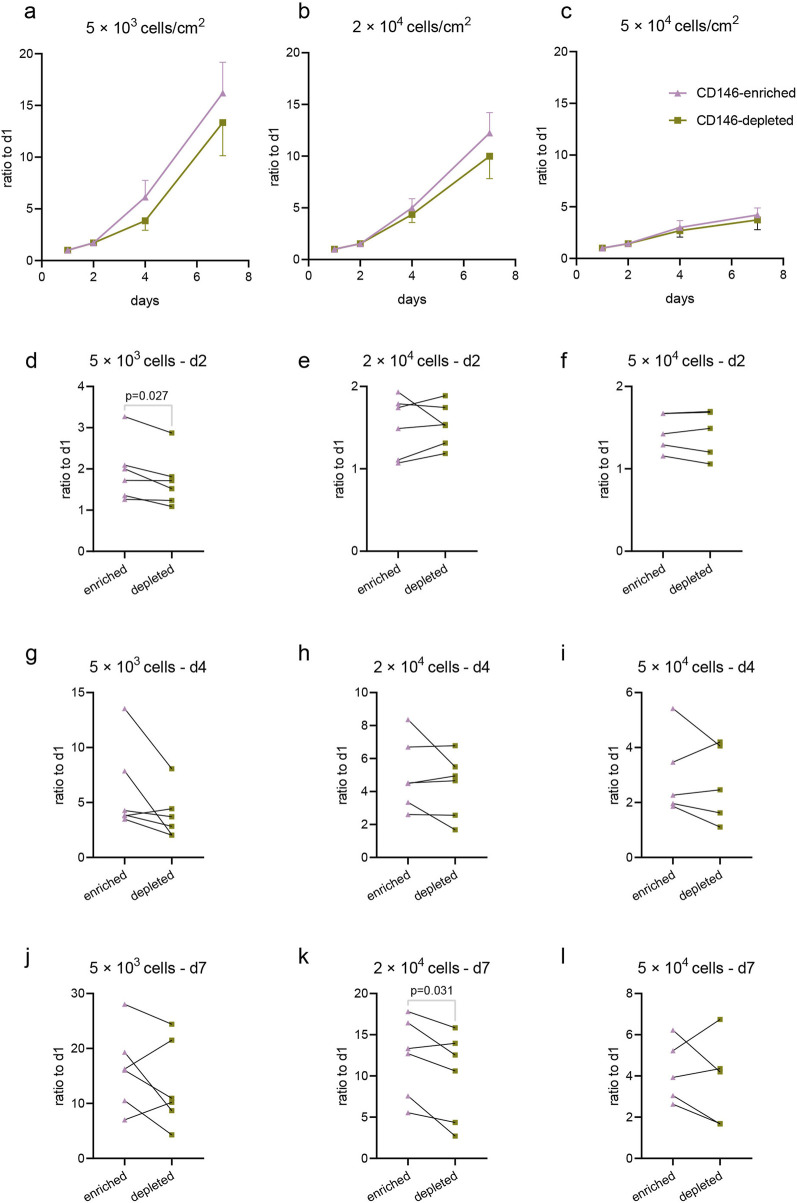
Proliferation of CD146-enriched and CD146-depleted subpopulations of hPDL-MSCs. CD146-enriched and CD146-depleted subpopulations were seeded onto cell culture plates at different densities: 5 × 10^3^ cells/cm^2^ (n = 6), 2 × 10^4^ cells/cm^2^ (n = 6), and 5 × 10^4^ cells/cm^2^ (n = 5). Cell viability was assessed with CCK8 at days 1, 2, 4, and 7 post-seeding, and the proliferation rates were calculated as a fold-over day 1 measurements of absorbance at 450 nm. Mean values and SEM from all donors are plotted as growth curves for seeding densities 5 × 10^3^ cells/cm^2^ **(A)**, 2 × 10^4^ cells/cm^2^ **(B)**, and 5 × 10^4^ cells/cm^2^ **(C)**. Respective densities are also presented individually on day 2 **(D–F)**, day 4 **(G–I)**, and day 7 **(J–L)**. Statistically significant results, determined with a paired t-test, are observed on graphs **(D, K)**.

Figure 6 shows the alterations in the viability of CD146-enriched and depleted subpopulations for cells seeded at different densities (5 × 10^3^, 2 × 10^4^, and 5 × 10^4^ cells per cm^2^) and cultured for 7 days. The highest increase in viability during culturing was observed in cells seeded at the lowest density, and the lowest increase in this parameter in cells seeded at the highest density (Figures 6A–C). Collectively, in cells seeded at the lowest density, CD146-enriched cells had a higher proliferation rate among almost all donors, with statistically significant results at the start of the experiment on day two (Figures 6D, G, J). In cells seeded at the middle density, faster proliferation of CD146-enriched cells was observed at a statistically significant level only at the end of the experiment on day seven (Figures 6E, H, K). No differences in proliferation rates between enriched and depleted subpopulations were observed in cells seeded at the highest density (Figures 6F, I, L). The data for individual donors are shown in Supplementary Figure 3.

**FIGURE 7:**
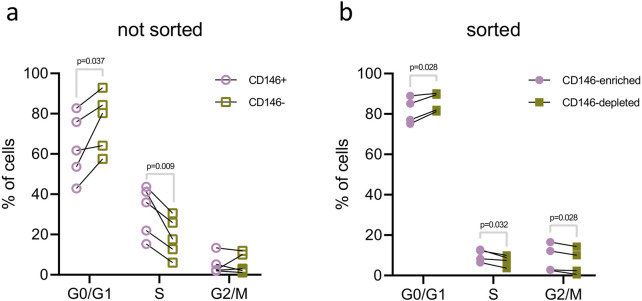
Cell cycle analysis of hPDL-MSCs with various rates of CD146+. The analysis of cell cycle was performed in the original hPDL-MSCs population **(A)** and isolated CD146-enriched and CD146-depleted subpopulations **(B)**. hPDL-MSCs from original populations (n = 5) were first stained with a monoclonal antibody for CD146, then DAPI for dsDNA, and examined with flow cytometry. First, CD146+ and CD146− cells were gated and dsDNA content was analyzed using histograms and the “Multicycle DNA” feature in FCS Express 7. Isolated CD146-enriched and CD146-depleted subpopulations of hPDL-MSCs (n = 4) were only stained for dsDNA with DAPI and analyzed in the same manner as described above. Statistical analysis was performed using paired t-tests.

The cell cycle analysis of the CD146+ and CD146− cells of original hPDL-MSCs, and isolated CD146-enriched and depleted subpopulations is shown in Figure 7. In the original hPDL-MSCs, the percentage of cells in the G0/G1 phase was significantly lower in CD146+ cells than in CD146− cells. Furthermore, the percentage of cells in the S phase was significantly higher in CD146+ cells than in CD146− cells (Figure 7A). Analysis of isolated CD146-enriched and depleted subpopulations showed a significantly lower percentage of enriched cells in the G0/G1 phase and a significantly higher percentage of enriched cells in the S and G1/M phases, compared to CD146-depleted subpopulation (Figure 7B).

## 4 Discussion

In this study, we investigated variability in the rate of CD146+ hPDL-MSCs, and how it might be regulated by various experimental conditions, such as cell culture density, passage, and exposure to inflammatory cytokines. Furthermore, we applied magnetic cell sorting and isolated two hPDL-MSCs populations with various levels of surface CD146+ rates. Then, we tested the basic properties of these populations, like proliferation and osteogenic differentiation. Our study identified several factors that might influence CD146 expression in hPDL-MSCs.

We found an extremely high heterogeneity in the surface CD146+ rate between different donors. Among 22 donors, the percentage of CD146+ hPDL-MSCs varied between 3% and 67% (Figure 2). The heterogeneity in rates of CD146+ cells among different donors has already been shown for hPDL-MSCs in previous studies (Xu et al., 2009; Iwasaki et al., 2013). The phenomenon of heterogeneity within MSCs populations is a topic that has been gaining interest lately, especially in the context of MSC-based therapies. Tissue source, donor-to-donor, and variation in preparation are commonly listed as sources of MSCs’ heterogeneity, also in regards to CD146 levels (Česnik and Švajger, 2024). Problems in preclinical and clinical studies arise from underreported MSC characteristics, culture conditions, and manufacturing processes (Renesme et al., 2022). In our study, we focused on the heterogeneity of CD146 in just one MSC source; however, even in that case, the source of this heterogeneity is unclear. CD146+ cells are mainly found in the perivascular space around blood vessels of a healthy human periodontal ligament (Chen et al., 2006), and have pericyte-like characteristics (Iwasaki et al., 2013). CD146 is a reliable marker for pericytes (Chen et al., 2018; Nanni et al., 2023), which allows us to draw the conclusion that variability in the rate of CD146+ cells between donors stems from differences in the composition of blood vessels. To further prove this hypothesis, it would be beneficial to perform a molecular-level investigation of underlying mechanisms regulating CD146 expression, for example regarding signaling pathways.

Having addressed tissue source and donor-to-donor variability, we focused our study on culture conditions as a source of CD146 heterogeneity. We showed that the percentage of CD146+ hPDL-MSCs declines after reaching confluency (Figure 3). The degree of the decline seemed to correlate with the initial cell seeding density, and how long cells were kept confluent in culture. Particularly, hPDL-MSCs seeded at the highest initial density reached the confluence earlier and exhibited the largest decline in the percentage of CD146+ cells. Previous studies already reported the influence of prolonged culture on CD146 expression. A recent study on hPDL-MSCs showed a rapid decline in the percentage of CD146+ cells after passage 7 (Alves et al., 2023). Another research on hPDL-MSCs observed a decline in mean fluorescence intensity of CD146 after passage 2 (Choi et al., 2015). One study on MSCs isolated from the human umbilical cord showed that decline in the percentage of CD146 cells inversely correlates with the telomere length, and this might account for the decline in the rate of CD146+ cells after several cell division cycles (Kouroupis et al., 2014).

We have further investigated the effect of various cytokines on the CD146 expression in hPDL-MSCs. We have focused on the effect of IL-1β, IFN- γ, and TNF-α for two reasons. First, these cytokines are involved in the progression of periodontitis, confirmed in saliva samples and gingival biopsies (Górska et al., 2003). IL-1β and TNF-α are connected with extracellular matrix degradation and alveolar bone resorption. An elevated level of IFN-γ in the periodontium has been linked to the severity of tissue inflammation in periodontal lesions, and the progression of tissue destruction in the periodontal ligament (Fiorillo et al., 2018; Sheibak et al., 2022). Second, these cytokines strongly enhance the immunomodulatory abilities of various MSCs, including those of hPDL-MSCs (Andrukhov et al., 2019). In particular, our previous studies showed that different cytokines activate distinct patterns of immunomodulatory proteins in hPDL-MSCs, which also results in qualitatively different immunomodulatory effects of these cells (Behm et al., 2019; Behm et al., 2020a; Behm et al., 2024a).

In our study, treatment of hPDL-MSCs with IL-1β and TNF-α increased the rate of CD146+ cells. In contrast, the treatment of hPDL-MSCs with IFN-γ resulted in a decline in the percentage of CD146+ cells (Figure 4). To the best of our knowledge, this is the first report on the effect of different cytokines on the rate of CD146+ cells in dental MSCs. Studies on other cell types are generally in agreement with our observation. Particularly, CD146 expression in human umbilical vein endothelial cells (HUVECs), pericytes, and blood endothelial cells was upregulated after stimulation with TNFα and IL-1β, presumably through NF-kB transactivation (Wang et al., 2013; Xing et al., 2014; Nanni et al., 2023). We have hypothesized that the alteration in CD146+ cells could be due to CD146 release from the cell surface, as it has been reported for cancer cells (Stalin et al., 2016) and endothelial cells (Boneberg et al., 2009). Therefore, we measured the content of CD146 in the conditioned media after different treatments (Supplementary Figure 1). However, our data did not prove this hypothesis.

As a further step, we isolated two populations of hPDL-MSCs with different rates of CD146+. We used magnetic sorting, which is a common method to quickly separate different cell populations with various surface-specific markers. With this isolation protocol, we were able to achieve distinct CD146-enriched and CD146-depleted hPDL-MSCs subpopulations, with 80% and 32% of CD146+ cells, respectively (Figure 5). The percentage of CD146+ cells in both populations did not change significantly over time in cell culture, albeit the difference between the subpopulations grew smaller after passaging. The CD146-enriched hPDL-MSCs declined in percentage of CD146+ cells, and CD146-depleted increased in CD146+ cells. Similar results were reported when hPDL-MSCs with higher and lower CD146+ rate were cultured for prolonged periods of time (Saito et al., 2013).

Previous studies investigating hPDL-MSCs used both fluorescence-activated cell sorting (FACS), and magnetic sorting to obtain cells with higher and lower CD146 expression; however, it is difficult to assess the efficiency, as the studies do not state the number of CD146+ and CD146− cells prior and post isolation. In our study, we decided to use magnetic separation, which is much faster compared to the FACS (Neurauter et al., 2007; Sutermaster and Darling, 2019), and still allows for the separation of the populations with different levels of CD146 expression and various functional properties. Moreover, using detachable magnetic beads creates less strain on the cell and prevents the influence of additional substances in downstream analysis. As CD146 is an adhesion molecule, we decided it would be important to detach the antibody from the cell surface after sorting, so that there would be no interference in CD146 expression coming from an extra element. Therefore, using the magnetic separation can be useful and reasonable for the application in the future.

hPDL-MSCs populations exhibited various functional properties depending on the surface rate of CD146+. Particularly, CD146-enriched subpopulations exhibited a faster proliferation rate than CD146-depleted subpopulations. This finding is in agreement with previous studies, which investigated proliferation rates through colony forming, cell counting, and CCK-8 assays (Saito et al., 2013; Zhu et al., 2013; Cho et al., 2016). In all cases, CD146-enriched subpopulations of hPDL-MSCs had faster proliferation than CD146-depleted subpopulations. To better understand the mechanism behind observed results, we compared the cell cycle between CD146-enriched and CD146-depleted hPDL-MSCs, and also CD146+ and CD146– cells from the original, not sorted hPDL-MSCs population. In both cases, CD146-enriched and CD146+ cells had a larger share of cells in the synthesis (S) phase and smaller in the gap 1 (G1) phase. This finding might account for the different proliferation rates of hPDL-MSCs with different rates of CD146+. Our results confirm previous cell cycle reports (Saito et al., 2013). Furthermore, CD146-enriched cells exhibited superior osteogenic differentiation potential, which is also in agreement with previous reports (Saito et al., 2013; Zhu et al., 2013; Cho et al., 2016). To investigate osteogenic potential, we used quantified Alizarin Red staining, which has been a gold standard for this purpose, and used in dental stem cells (Stanford et al., 1995; Camassari et al., 2023). Due to the possibility of using hPDL-MSCs in periodontal ligament regeneration, we focused our research mainly on osteogenic differentiation. With the proximity of PDL to the alveolar bone, adipogenic and chondrogenic differentiation would be of lesser importance.

For clinical applications, the topic of appropriate cell expansion has been continuously raised as a potential issue for the therapeutic success of dental MSCs (Bansal and Jain, 2015; Tatullo et al., 2024), and in particular hPDL-MSCs (Zhu and Liang, 2015). Based on our observations, the appropriate culturing methods have to be implemented in order to maintain surface CD146 expression. It has been suggested that the heterogeneity of CD146+ rate in dental MSCs could originate from their *in vitro* expansion (Chen et al., 2022), as CD146 expression could gradually decrease with passages and proliferation (Li et al., 2023). Our results confirm this statement, and highlight initial seeding density and inflammatory cytokine stimulation as factors influencing the CD146+ rate in hPDL-MSCs. As CD146+ hPDL-MSCs were proven to have superior qualities, we believe that maintaining CD146 expression will be beneficial for cells used in regenerative dentistry.

The effectiveness of CD146+ hPDL-MSCs in clinical applications is still to be proven. These cells are more proliferative and have higher osteogenic potential. Those qualities could also matter in other novel approaches to periodontal diseases treatments, such as epigenetic reprogramming (Li et al., 2024). However, it is known that transplanted MSCs exert their effect by secreting the trophic factors and creating a favorable environment and immunomodulation rather than differentiation into functional cells (Wang et al., 2014; Andrukhov et al., 2019; Han et al., 2022). Further studies investigating differences in intrinsic properties between CD146+ and CD146− cells are required, particularly focusing on differences in signaling pathways (e.g., Wnt, Notch, or TGF-β) and immunomodulatory abilities. Aside from the direct medical use, hPDL-MSCs (and other MSCs) can also be used in creation of 3D cell culture models, which help with improvement of dental care and development of new therapies (Y Baena et al., 2022). The results of our experiments can be implemented in such model creation.

### 4.1 Limitations

Our study explored the influence of culturing conditions on hPDL-MSCs, therefore the greatest care was put into limiting impact of subsequent experimental runs. However, it is not feasible to completely exclude the possibility of some small changes, for example in reagent composition from lot to lot. Another limitation of our study is the presence of CD146− cells in CD146-enriched subpopulation, and CD146+ in CD146-depleted subpopulation. Cleaner separation was not possible without the use of other techniques, such as FACS or knock-down. Nevertheless, with a cut off 30% difference in CD146+ rate, we were still able to detect functional differences between the populations. Additionally, magnetic bead separation proved to be a reliable, fast, and relatively cheap method to isolate CD146-enriched and CD146-depleted hPDL-MSCs, which does not require additional equipment. In our study, we used cells from donors in a limited age range. However, investigating the effects of age on CD146 expression was not the purpose of our study, hence those relations were not explored. As this is a 2D *in vitro* study, it would be beneficial to follow it up with a 3D model or an *in vivo* investigation.

### 4.2 Conclusion

CD146 is a marker of hPDL-MSCs with enhanced proliferative capacities and osteogenic potential. The rate of CD146+ cells is influenced by culture conditions and inflammatory stimuli. With this publication, we want to highlight the importance of proper and optimized culturing techniques for MSCs’ expansion and priming to be used in cell therapies. Further research on CD146 regulation will advance the understanding of MSCs’ biology and improve their therapeutic potential.

## Data Availability

The original contributions presented in the study are included in the article/Supplementary Material, further inquiries can be directed to the corresponding author.
