# Supplementary material for: Regulation and functional importance of human periodontal ligament mesenchymal stromal cells with various rates of CD146+ cells
Source: Front Cell Dev Biol. 2025 Mar 7;13:1532898. doi: 10.3389/fcell.2025.1532898 (PMC11925893; doi:10.3389/fcell.2025.1532898)
Supplement: Supplementary file 1 [file DataSheet1.docx]

Supplementary Material

**Supplementary Table 1. List of donors used for experiments.**

hPDL-MSCs from 22 donors were used in all experiments. The table lists all donors used for each experiment, the sex of the donor, and which passage was used in each figure. “no” stands for number, “fig” stands for figure, “sup fig” stands for supplementary figure.

| **order no** | **sex** | **fig 2** | **fig 3** | **fig 4** | **fig 5 b-c** | **fig 5 e** | **fig 6** | **fig 7** | **sup fig 2** | **sup fig 3** |
| --- | --- | --- | --- | --- | --- | --- | --- | --- | --- | --- |
| **1** | Female | 4 | - | - | - | 6 | - | - | - | - |
| **2** | Male | 6 | - | - | - | - | 5 | - | - | - |
| **3** | Female | 5 | - | - | - | - | 5 | - | - | - |
| **4** | Male | 6 | - | 6 | 5 | - | - | 5 | 6 | 5 |
| **5** | Male | 4 | - | - | - | - | 4 | - | - | - |
| **6** | Male | 5 | - | 5 | - | 7 | 4 | - | - | - |
| **7** | Female | 7 | - | 7 | - | 7 | 5 | - | - | - |
| **8** | Female | 4 | - | - | - | - | 4 | - | - | - |
| **9** | Male | 7 | - | 7 | 7 | - | 5 | 8 | - | 7 |
| **10** | Female | 6 | - | 5 | - | - | - | - | 5 | - |
| **11** | Female | 6 | - | 5 | - | - | - | - | 5 | - |
| **12** | Female | 4 | - | 6 | - | - | 5 | - | 6 | - |
| **13** | Male | 5 | 5 | 6 | 7 | - | - | 7 | - | 7 |
| **14** | Female | 6 | 5 | - | - | 6 | 4 | - | - | - |
| **15** | Female | 6 | 6 | 5 | 7 | 7 | - | 7 | - | 7 |
| **16** | Female | 7 | - | 6 | 7 | - | - | - | 6 | 7 |
| **17** | Male | 7 | - | 6 | - | - | - | 7 | 6 | - |
| **18** | Female | 7 | - | 7 | - | - | - | - | - | - |
| **19** | Male | 5 | - | 7 | - | - | - | - | - | - |
| **20** | Female | 4 | - | 7 | - | - | - | - | - | - |
| **21** | Male | 5 | 5 | 5 | - | - | - | - | - | - |
| **22** | Female | 5 | - | 4 | 6 | - | - | 6 | - | 6 |


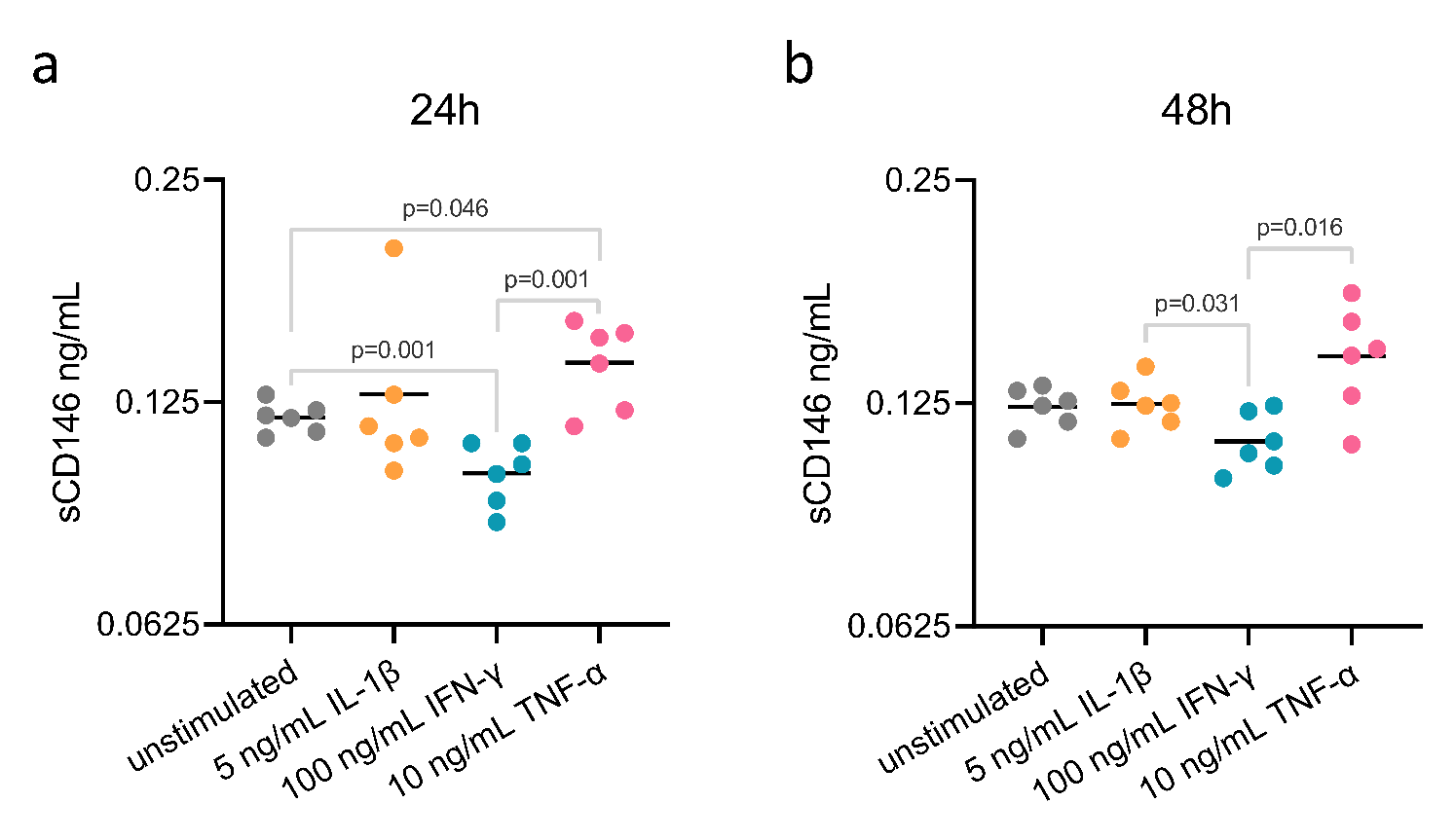


**Supplementary Figure 1.** **CD146 levels in conditioned media of hPDL-MSCs treated with inflammatory cytokines.**

hPDL-MSCs from 5 donors were treated with inflammatory cytokines: IL-1β (5 ng/mL), IFN-γ (100 ng/mL), and TNF-α (10 ng/mL) for either 24 hours (**a**) or 48 hours (**b**). Afterward, conditioned media were collected, and CD146 concentration levels were determined using Human CD146 ELISA. The results were obtained by measuring the absorbance at 450 nm and calculating the concentrations based on the extrapolated standard curve. Each dot within a group presents a different donor (n=6), with mean values represented by the line. Statistical significance was determined with RM one-way ANOVA and post-hoc Tukey.


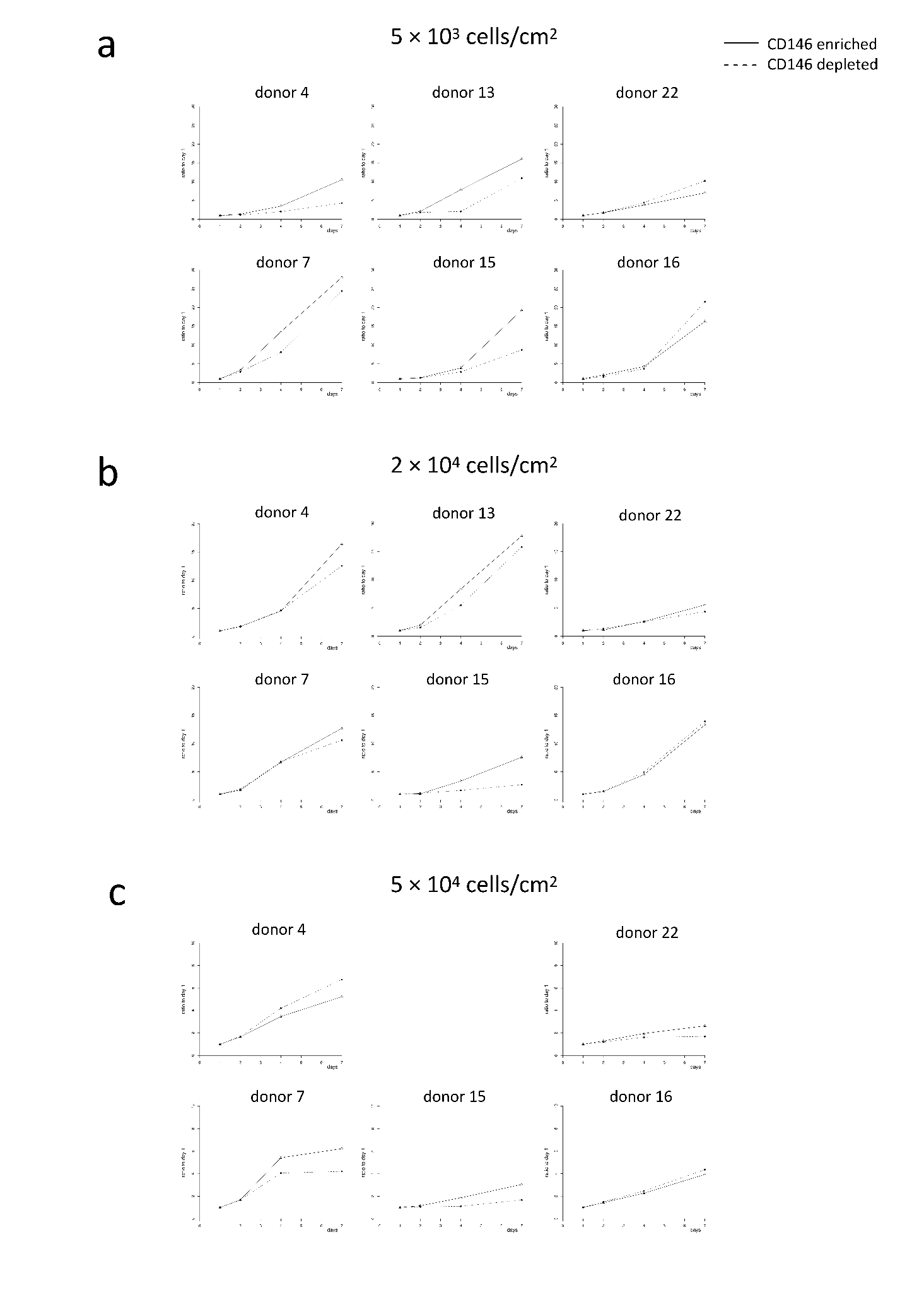


**Supplementary Figure 2.** **Proliferation of CD146-enriched and CD146-depleted subpopulations of hPDLMSCs in individual donors.**

The growth curves present CD146-enriched and CD146-depleted subpopulations seeded onto cell culture plates at different densities: 5 × 10^3^ cells/cm^2^ (**a**), 2 × 10^4^ cells/cm^2^ (**b**), and 5 × 10^4^ cells/cm^2^ (**c**). Cell viability was assessed with CCK8 at days 1, 2, 4, and 7 post seeding, and the proliferation rates were calculated as fold-over day 1 measurements of absorbance at 450 nm. Each point represents a technical triplicate measurement. The growth curves show variability among individual donors.
